# Degeneration-Dependent Retinal Remodeling: Looking for the Molecular Trigger

**DOI:** 10.3389/fnins.2020.618019

**Published:** 2020-12-18

**Authors:** Michael Telias, Scott Nawy, Richard H. Kramer

**Affiliations:** ^1^Department of Molecular and Cell Biology, University of California, Berkeley, CA, United States; ^2^Helen Wills Neuroscience Institute, University of California, Berkeley, CA, United States

**Keywords:** retinal degeneration, retinal remodeling, synaptic plasticity, retinoic acid, retinal ganglion cell, retinitis pigmentosa, age-related macular degeneration

## Abstract

Vision impairment and blindness in humans are most frequently caused by the degeneration and loss of photoreceptor cells in the outer retina, as is the case for age-related macular degeneration, retinitis pigmentosa, retinal detachment and many other diseases. While inner retinal neurons survive degeneration, they undergo fundamental pathophysiological changes, collectively known as “remodeling.” Inner retinal remodeling downstream to photoreceptor death occurs across mammalian retinas from mice to humans, independently of the cause of degeneration. It results in pervasive spontaneous hyperactivity and membrane hyperpermeability in retinal ganglion cells, which funnel all retinal signals to the brain. Remodeling reduces light detection in vision-impaired patients and precludes meaningful vision restoration in blind individuals. In this review, we summarize current hypotheses proposed to explain remodeling and their potential medical significance highlighting the important role played by retinoic acid and its receptor.

## Introduction

The retina is a multilayered sensory organ located in the back of the eye, in the inner side of the eyecup, behind the lens (Kolb, 1995). It is divided into two main compartments: the outer retina, populated by photoreceptors (rods and cones), and the inner retina, populated by neural and glial cells, including horizontal cells, bipolar cells, amacrine cells, retinal ganglion cells (RGCs), astrocytes and microglia. Large retinal-specific cells, known as Müller glia, span radially across the outer and inner retinal regions. Light stimulation of photoreceptors initiates a chain of electrochemical responses which are transmitted and modulated by retinal interneurons (i.e., horizontal, bipolar, and amacrine cells), finally converging on RGCs. RGCs reside in the ganglion cell layer (GCL), the inner-most layer of the retina, and their axons form the optic nerve, which leaves the retina and enters the brain, synapsing at the lateral geniculate nucleus on both hemispheres. From there, visual information is finally sent into the primary visual cortex area (V1) located in the occipital lobe. The basic retina-to-brain pathway of visual information is shared by virtually all known mammals as well as other animals with anatomical parallels.

In humans, most cases of vision loss are due to photoreceptor degeneration. Photoreceptor cell death can be the result of many different types of diseases and disorders, including inherited monogenic retinitis pigmentosa (RP, incidence ∼1:4000) (Verbakel et al., 2018), idiopathic age-related macular degeneration (AMD, ∼200 million patients worldwide) (Wong et al., 2014), retinal detachment (Kreissig, 2016), and environmental injuries (Athanasiou et al., 2013).

In RP rods die before cones, night-vision is lost early-on, and the visual field increasingly shrinks from the periphery to the center, until all photoreceptors die. The process can take years to decades in humans and weeks to months in different animal models (Collin et al., 2020; Winkler et al., 2020). Many different mutations in several different genes can cause RP. If the mutation is characterized early on, gene-therapy mediated delivery of the corrected gene can prevent or reduce photoreceptor death (Miraldi Utz et al., 2018; Takahashi et al., 2018). However, in most cases, treatment is unfeasible, and only a handful of successful clinical trials have been approved and conducted so far. Therefore, RP still remains an orphan disease with no routine treatment that can slow down photoreceptor loss or improve residual vision before complete blindness. Transgenic mice and rats with single-gene mutations, for example in Rhodopsin or Pde6b, reliably recapitulate the features of the disease in humans, with progressive vision loss ending in total absence of photoreceptors and light responses (Nakazawa et al., 2019). In addition, there are also important RP models in large mammalians, including dogs, cats, sheep, horses, pigs and non-human primates (Winkler et al., 2020).

As opposed to RP, AMD is much more frequent and caused by a still unknown etiology, even though specific risk factors have been identified (Hageman et al., 2008). Cones degenerate in the macula, where their density is the highest, at the point of sharpest vision in the human retina. High-acuity vision is damaged, and the central visual field is affected much more than the periphery. An initial phase of this disease, called dry-AMD, is characterized mainly by the death of photoreceptor cells. It, is followed by wet-AMD, characterized by expanding deterioration of the retinal tissue, including neovascularization and the formation of drusen deposits. Neovascularization is driven primarily by an increase in vascular-endothelial growth factor (VEGF). Most available anti-AMD treatments are designed to reduce or slow-down neovascularization, using monoclonal antibodies against VEGF or its receptor (Gil-Martinez et al., 2020). However, there is currently no treatment that can prevent or stop the death of photoreceptors, let alone reverse it. The macula is a sub-compartment of the retina which, in mammalians, exists only in primates. Therefore, available animal models used for AMD research, from rodents to pigs and rhesus monkeys, can recapitulate only some aspects of the disease (Zeiss, 2010; Pennesi et al., 2012).

## Downstream Effects of Photoreceptor Degeneration

The progressive death of photoreceptors in the outer retina sends ripple effects to the much less numerous inner retinal neurons, including most critically the RGCs, which funnel all visual information out of the retina. In response to the progressive death of photoreceptors, there are a number of both structural and functional changes in inner retinal neurons and RGCs. These pathophysiological changes, observed in the surviving inner retina cells, are collectively known as remodeling (Jones et al., 2012, 2016a), and have been described in human AMD (Jones et al., 2016c) and human RP (Jones et al., 2016b). Structural changes in the surviving areas include *de novo* neuritogenesis and dendritic reorganization, cell migration and layer disruption, and the formation of a thick inner limiting membrane by reactive Müller glia cells (Iandiev et al., 2006; Lin et al., 2012a; Pfeiffer et al., 2020). Functional abnormalities across outer and inner retinal circuits include changes in synaptic transmission and electrical coupling among similar and different neuronal subtypes, as well as persistent spontaneous oscillations and retinal waves (Marc et al., 2007; Lin et al., 2012b; Euler and Schubert, 2015). RGCs develop intrinsic spontaneous hyperactivity and cell membrane hyperpermeability (Tochitsky et al., 2016; Telias et al., 2019; Denlinger et al., 2020).

These structural and functional abnormalities observed during remodeling do not happen in unison across the degenerating retina. Changes such as neuronal hyperactivity and enhanced membrane hyperpermeability can be observed as soon as days or weeks upon degeneration onset in mice and rat models of RP and AMD (Tochitsky et al., 2016; Telias et al., 2019; Denlinger et al., 2020), while structural changes and the disruption of cell layers happen only upon prolonged degeneration of many weeks to months (Garcia-Ayuso et al., 2019; Pfeiffer et al., 2020). Collectively, functional remodeling results in a “noisy” retina, obscuring remaining light-responses and reducing signal fidelity in treatments aimed at restoring vision (Barrett et al., 2015; Stasheff, 2018).

## Retinoic Acid-Dependent Signaling Is Essential for Remodeling

Every day, rod and cone cells can synthesize millions of copies of opsin proteins and there are hundreds of millions of rods and cones in the mouse retina (Kolb, 2005; Goldberg et al., 2016). To react to light, each individual opsin molecule binds to retinaldehyde (specifically, *11-cis-retinal*), the universal chromophore (Daruwalla et al., 2018). Retinaldehyde in mammals is synthesized by dehydrogenation of vitamin A (retinol), a reaction carried out by retinol dehydrogenase (RDH) in RPE cells, rods, cones and Müller glia cells. In humans suffering from malnutrition, vitamin A deficiency results in night blindness and photoreceptor cell damage and subsequent degeneration (Wiseman et al., 2017). Retinaldehyde is oxidized into retinoic acid (RA) by retinaldehyde dehydrogenase (RALDH), an enzyme that is expressed across retinal cells (Luo et al., 2006; Harper et al., 2015). Binding of RA to its nuclear receptor (RAR_α/β/γ_) releases it from co-repressor (CoR), allowing it to form a heterodimeric complex with retinoid orphan receptors (RXR_α/β/γ_) that can bind DNA and act as a transcriptional activator and enhancer.

An early *ex vivo* study of retinal bipolar cells, testing the hypothesis that RA is the trigger behind remodeling, was conducted using light-induced retinal degeneration in albino mice (Lin et al., 2012a). The effects of remodeling were assessed by measuring neuritogenesis in bipolar cells. *De novo* neuritogenesis and an increase in neurite length shown in degenerated retinas, were recapitulated when RA-signaling was enhanced. Conversely, they were prevented when RA-signal was pharmacologically or genetically blocked. Interestingly, manipulation of RXR had a stronger effect on remodeling than similar manipulations of RA synthesis and RAR activity. Retinal degeneration was associated with increased translation of glutamate receptor 2 (GluA2) and β-Ca^2+^/calmodulin-dependent protein kinase II (βCaMKII) in the inner retina, where all three RXR isoforms directly bind βCaMKII during remodeling. This might suggest a form of homeostatic plasticity in response to increased excitation. However, the molecular mechanism downstream to this interaction, and how it is part of neuritogenesis, or any other aspect of remodeling, was not elucidated. Furthermore, in a model of light-induced retinal damage, neuritogenesis is observed as soon as a few days after insult, while in most transgenic mouse and rat models of RP, these changes occur during late stages of remodeling, but hyperactivity can be detected very early-on. Thus, although morphological reorganization of dendrites in the inner plexiform layer might have important consequences to signal processing and synaptic plasticity, it is not likely to be the source of persistent retinal hyperactivity and sustained spontaneous electrical oscillations, but rather its result.

Our studies have demonstrated that RA/RAR-signaling is necessary and sufficient to explain two critical and early aspects of remodeling in RGCs: hyperactivity and hyperpermeability (Tochitsky et al., 2016; Telias et al., 2019; Denlinger et al., 2020). Using mouse models for fast and slow RP (rd1 and rd10, respectively), we found that photoreceptor degeneration is directly associated with a P2X-dependent increase in RGCs membrane permeability. This increase in permeability, termed hyperpermeability, was measured by *ex vivo* incubation of retinas with nuclear dyes that can pass through the P2X receptor pore (“Yo-Pro”) but are otherwise unable to enter cells. In wildtype retinas only ∼5% of all GCL cells (excluding vascular pericytes) loaded Yo-Pro, while ∼30% of cells did so in rd retinas. Loading was always prevented by pre-incubation with the P2X receptor antagonist TNP-ATP, and it could not be potentiated by adding the P2X receptor agonist Bz-ATP in neither type of retina. Hyperpermeability could be induced in healthy retinas when intravitreally injected with all-*trans* RA and was reduced in rd retinas upon intraocular injection of RALDH blockers or RAR antagonists. In wildtype mice, overexpression of a constitutively active form of RARα that is never repressed by CoR and does not require activation by RA, resulted in increased membrane permeability in almost every transduced RGCs. Furthermore, delivery of a dominant-negative form of RARα to rd mice prevented Yo-Pro permeability in RGCs. Therefore, we found RAR activation to be necessary and sufficient to induce P2X-dependent membrane hyperpermeability, in an ATP-independent manner.

**FIGURE 1:**
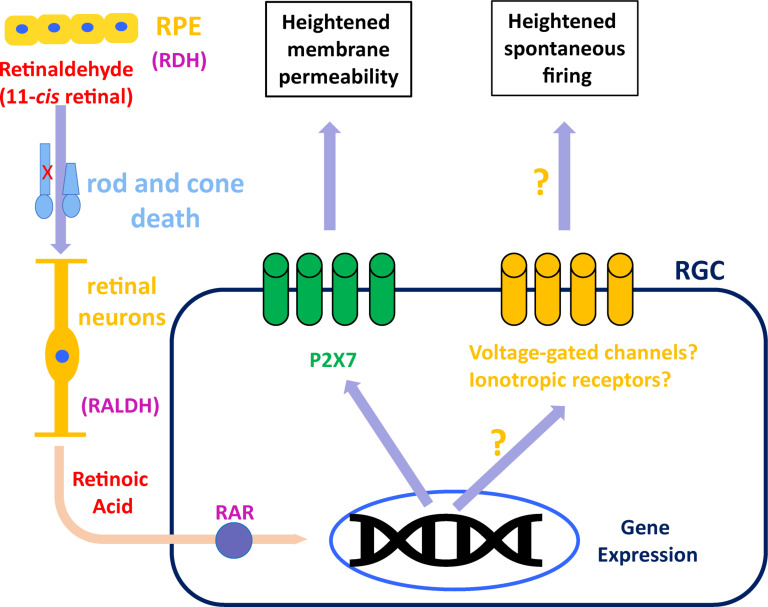
Suggested model for RAR-dependent remodeling. Retinaldehyde is synthesized in the outer retina by retinol dehydrogenase (RDH) in rods, cones and retinal pigmented epithelium (RPE). Excess retinaldehyde as the result of photoreceptor death is converted by retinaldehyde dehydrogenase (RALDH) into retinoic acid, probably in different retinal neurons and eventually transported to retinal ganglion cells (RGCs). In RGCs, retinoic acid binds its receptor (RAR), initiating transcriptional activation, which leads to increased P2X7 expression, making it hyperpermeable. Another, still unknown mechanism, results in RAR-dependent hyperactivity, probably via overexpression or activation of voltage-gated ion channels or ionotropic receptors for neurotransmitters.

Blockade of RAR activation in the inner retina of rd mice, using small-molecule inhibitors, resulted in reduced spontaneous hyperactivity 3–7 days later (Telias et al., 2019). Ablation of endogenous RAR activation by viral transduction of rd RGCs with dominant-negative RARα for 1–2 months, also resulted in decreased hyperactivity. Both treatments reduced the average frequency firing of RGCs by ∼50%, from 6–8 Hz to 2–4 Hz. Overall, activity levels in all cases were not affected by acute pharmacological blockade of synaptic input, suggesting that RGCs are the intrinsic and main source of hyperactivity during degeneration, as well as RA’s main cellular target. Given the way retinaldehyde and RA are transported between cells and shuttled from the cytoplasm to the nucleus (Maden, 2002), it might be the case that the source of RA to RGCs are Müller glial cells which take up retinaldehyde from the outer retina and re-distribute it throughout the inner retina, including the RGCs, where sustained RAR activation causes persistent post-synaptic hyperactivity (Figure 1).

Whatever mechanism delivers RA to RGCs, the resulting remodeling is stereotypical and found in RGCs of mice and rat models of RP (Tochitsky et al., 2016; Telias et al., 2019), as well as in a rat model of AMD (Denlinger et al., 2020). In RP mice, degeneration-dependent spontaneous hyperactivity obscures remaining light responses, and blocking RAR activation *in vivo* improves light sensitivity and visual perception (Telias et al., 2019). In AMD rats, RAR-dependent hyperpermeability in RGCs allows region-specific visual restoration using photoswitches (Denlinger et al., 2020). Since the P2X7 promoter does not include RARE sequences, it is unlikely that RAR directly drives an increase in P2X7 expression. Thus, factors that mediate the interaction between RAR-dependent transcription and P2X receptors, and whether the same factors are in charge for both hyperpermeability and hyperactivity, remain to be determined. Still, blocking RALDH, RA, or RAR is a promising and available prospective to augment residual vision in patients with slow retinal degeneration, and boost visual restoration in completely blind patients, by increasing the signal-to-noise ratio in RGCs.

## Synaptic Remodeling and Hyperactivity in Retinal Degeneration

The question of whether photoreceptor degeneration alters the circuitry in the outer and inner retina is critical for vision restoration. If the circuits remain unaltered, or any alterations to them are compensated for by a homeostatic counter-mechanism, then vision restoration technologies in humans, such as prosthetic devices, can be expected to achieve high acuity. Early studies on retinal remodeling showed that the surviving retina does not remain intact (i.e., spontaneous hyperactivity), and that these pathophysiological changes could be attributed to abnormal synaptic plasticity, including cell- and layer-specific alterations to the expression of metabotropic and ionotropic glutamate receptors in bipolar cells, amacrine cells and RGCs of degenerating retinas (Marc et al., 2003, 2007; Stasheff, 2008). Yet, other studies make the argument that the structure and function of the circuitries that converge on RGCs remain largely unchanged by photoreceptor degeneration (Margolis et al., 2008; Mazzoni et al., 2008).

A change in synaptic input can be indirectly measured by assessing the expression of synaptic proteins and neurotransmitter-associated receptors and enzymes, in inner retinal neurons in correlation with photoreceptor degeneration. When mice were subjected to light-induced retinal damage, photoreceptor death was associated with an increase in the expression and trafficking of ionotropic glutamate receptors (GluR2) in the outer plexiform layer 7 days later, but this increase subsides after 30 days (Lin et al., 2012b). Immunohistochemistry analysis of synaptic markers (i.e., RIBEYE and gephyrin) in WT vs. rd1 retinas, shows a decrease in excitatory input on ON-RGCs and an increase in GABAergic input onto OFF-RGCs in correlation with degeneration, but the distribution of the synaptic connections across the dendritic trees of RGCs remained unchanged by degeneration and the differences between WT and rd1 are relatively modest (Saha et al., 2016).

Photoreceptors are continuously releasing glutamate onto counterpart bipolar cells, and light reduces glutamate release at the ribbon synapse. As photoreceptors die, they lose their outer segments and therefore their reactivity to photons, leading to an expected loss of light-dependent changes in glutamate release in the outer retina, affecting bipolar cells as degeneration progresses. This sustained abnormal excitation of bipolar cells by dying photoreceptors would be expected to induce different downstream effects onto different types of amacrine cells and RGCs, depending on the ON or OFF sign of the second synapse. Indeed, studies into degeneration-dependent hyperactivity have shown that two separate oscillatory networks exist (Euler and Schubert, 2015). The oscillatory network in the outer retina, composed by dying cones, rod bipolar cells and horizontal cells, oscillates at a frequency of up to ∼3 Hz (Haq et al., 2014). These oscillations, the study claims, arise from retrograde release of GABA from horizontal cells back onto cones. The oscillatory network of the inner retina is composed by AII amacrine cells and cone bipolar cells, and oscillates at ∼10 Hz (Borowska et al., 2011; Trenholm et al., 2012). These inner retinal oscillations seem to be an important component of the spontaneous hyperactivation induced by photoreceptor death and were shown to be insensitive to blockers of voltage-gated Ca^2+^-channels and enhanced by blockers of hyperpolarization-activated currents (I_*h*_). They seem to originate from the activation of voltage-gated Na^+^-channels expressed in AII amacrine cells and spread to ON-bipolar cells through gap junctions. In particular, the role of gap junctions in mediating these oscillations has been confirmed in several studies (Ivanova et al., 2016). This is functionally meaningful, as in visually impaired rd mice *ex vivo* treatment with gap junction blockers reduces hyperactivity and improves light responses (Toychiev et al., 2013; Barrett et al., 2015).

However, these studies do not identify the trigger mechanism behind these plastic events in inner retinal neurons, and how these cells are able to “sense” the death of photoreceptors in the outer retinal compartment. While these hypotheses might explain many of the electrophysiological abnormalities associated with pathological hyperactivity, it is still insufficient to explain other aspects of remodeling, such as membrane hyperpermeability, *de novo* neuritogenesis and layer disruption. For specific synaptic proteins to increase or decrease their expression in specific cell types, a complex feedback signaling network must exist, ultimately triggering aberrant gene expression. Yet, these signaling networks and their “core” gene-expression event remains absent from studies into synaptic remodeling. Furthermore, in both mice and rats with retinal degeneration, blockade of all synaptic input onto RGCs is not sufficient to significantly reduce degeneration-dependent hyperactivity (Sekirnjak et al., 2009; Tochitsky et al., 2016; Telias et al., 2019; Denlinger et al., 2020). Most importantly, whatever functional and synaptic changes might be affecting different cell types across the outer and inner retina during degeneration, the structural constrains of the retina itself compel research to focus on the net effect of remodeling on RGCs, as all information leaving the retina must transit through them. While many studies show pathological or homeostatic plasticity taking place in bipolar and other cells, evidence from RGCs suggesting a significant pre-synaptic mechanism driving their hyperactivity is still missing at large. From this we conclude that, in order to reduce hyperactivity and boost residual light responses and visual restoration techniques, efforts must be put into understanding the mechanisms behind intrinsic pathophysiological changes caused by degeneration in RGCs.

## Summary and Conclusions

Finding the molecular mechanism behind pathophysiological remodeling is essential to block degeneration-dependent hyperactivity. During retinal degeneration, a process that can take decades in humans, remnant light responses are obscured by hyperactivity. Blocking or reducing hyperactivity unmasks light responses, opening a therapeutic window to enhance vision in patients suffering from RP, AMD and other photoreceptor degeneration diseases. In patients that are completely blind, restoration of vision might be possible through photo-pharmacology (Tochitsky et al., 2018), optogenetics (Baker and Flannery, 2018) or prosthetic devices (Mirochnik and Pezaris, 2019). However, low signal-to-noise ratio will result in poor visual restoration, regardless of the method employed. In the best-case scenario, the dead outer retina would be repopulated by implantation of photoreceptors differentiated from stem cells (Llonch et al., 2018). Yet, even in such a case, remodeling might persist, as its long-lasting effects and their reversibility are still largely unknown. Therefore, understanding the source of hyperactivity becomes critical.

As photoreceptors die, their necrotic outer disks and bodies release their content into the extracellular matrix. Since photoreceptors greatly outnumber the rest of the cells in the retina (∼70% of the cells in a mouse retina), their slow degeneration provides a sustained source of bioactive molecules that can percolate the inner retina, crossing cell and layer membranes. Among these, purines and retinoids have been proposed as candidates (Aplin et al., 2016; Telias et al., 2019). In parallel, loss of photoreceptors deprives bipolar cells of glutamate, triggering consequent pathological homeostatic mechanisms (Haq et al., 2014). All of these might contribute to the many aspects of remodeling, and most importantly to the pervasive hyperactivity that develops in the inner retina in general and in RGCs in particular.

## Author Contributions

MT, SN, and RK wrote and revised the manuscript. RK provided funding. All authors contributed to the article and approved the submitted version.

## Conflict of Interest

The authors declare that the research was conducted in the absence of any commercial or financial relationships that could be construed as a potential conflict of interest.
